# Supplementary material for: Age-Dependent Effects of Oxytocin and Oxytocin Receptor Antagonists on Bladder Contractions: Implications for the Treatment of Overactive Bladder Syndrome
Source: Biomedicines. 2024 Mar 18;12(3):674. doi: 10.3390/biomedicines12030674 (PMC10968663; doi:10.3390/biomedicines12030674)
Supplement: Supplementary file 1 [file biomedicines-12-00674-s001.zip › biomedicines-2878411-supplementary.pdf]

Supplementary Figures

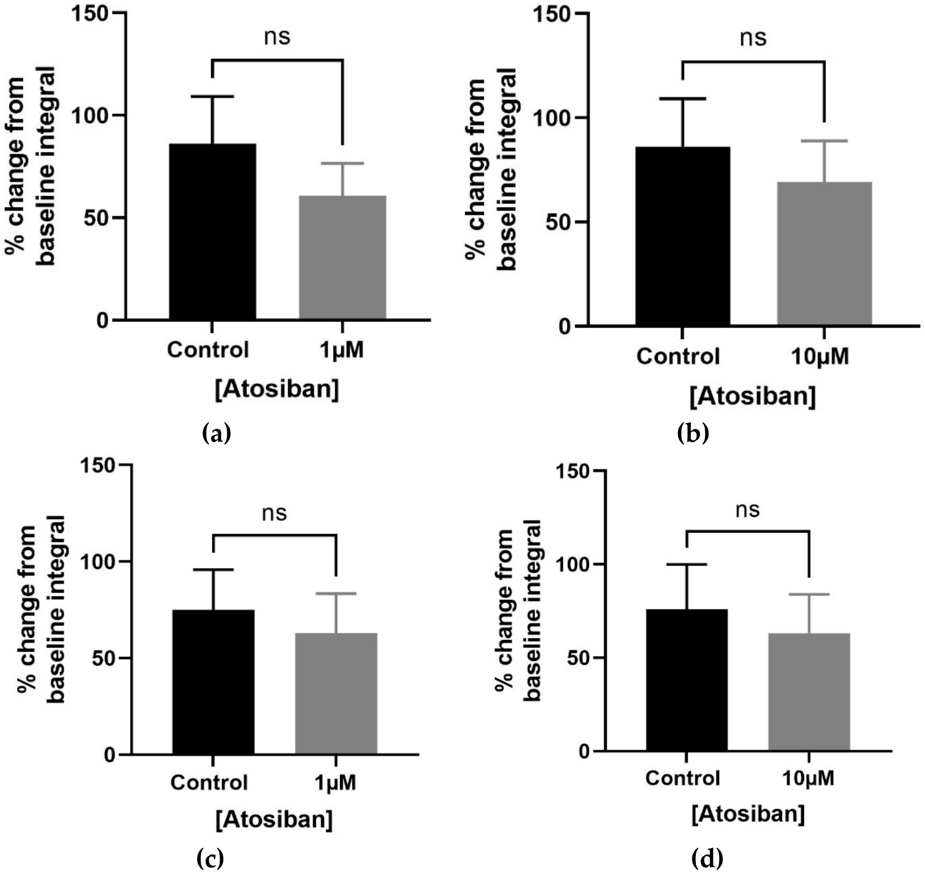

Figure S1. Effects of exogenous atosiban on bladder contractions. Rat bladder was incubated with both low (1 µM) and high (10 µM) concentrations of atosiban-containing organ baths, leading to a decrease in baseline integral parameters of spontaneous contractions in both older (7–9 months) (a & b) and young (7–8 weeks) (c & d) rats, respectively, measured relative to the maximum percentage of KCl (20 mM) [unpaired t-test, $n = 5$, $p \geq 0.05$] (ns indicates non-significant effect).

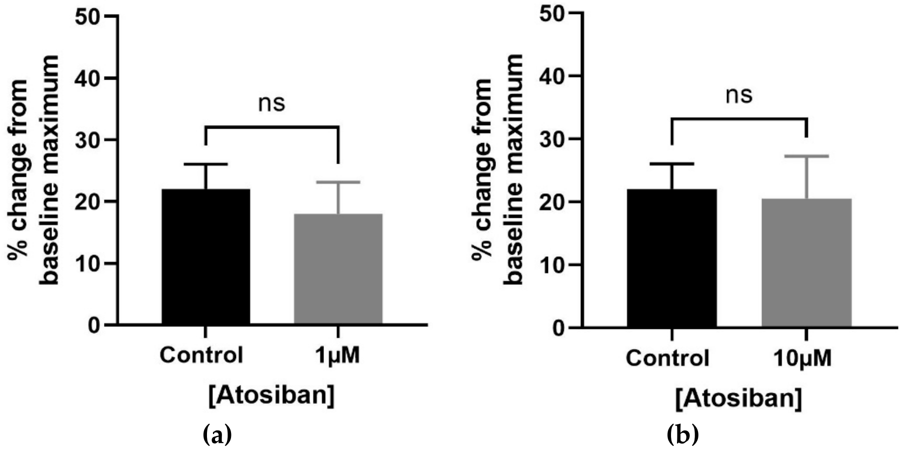

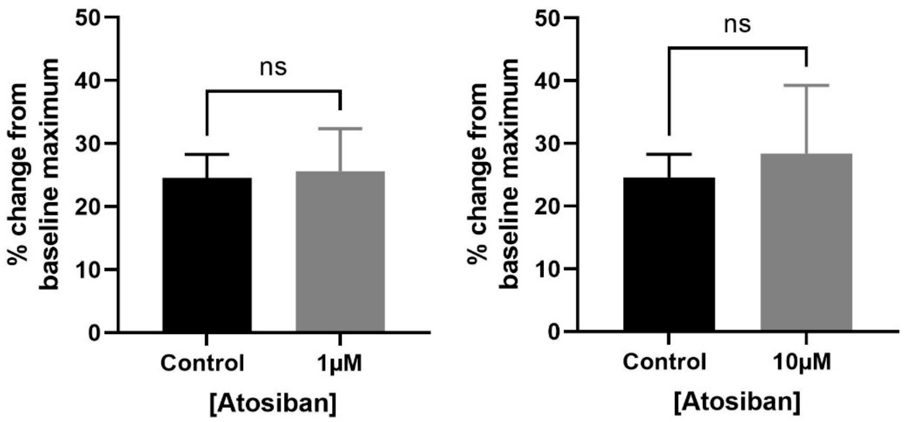

(c) (d)

Figure S2. Comparison of the effects of atosiban on bladder contractions. Atosiban, at a concentration of 1 µM & 10 µM, showed a decrease in the % change in baseline maximum parameter (non-significant) of spontaneous contractions in older (7–9 months) (a & b), but not young (7–8 weeks) (c & d) rats, averaged relative to the maximum percentage of KCl (20 mM) [unpaired t-test, *n* = 5, $p \geq 0.05$] (ns denotes non-significant effect).

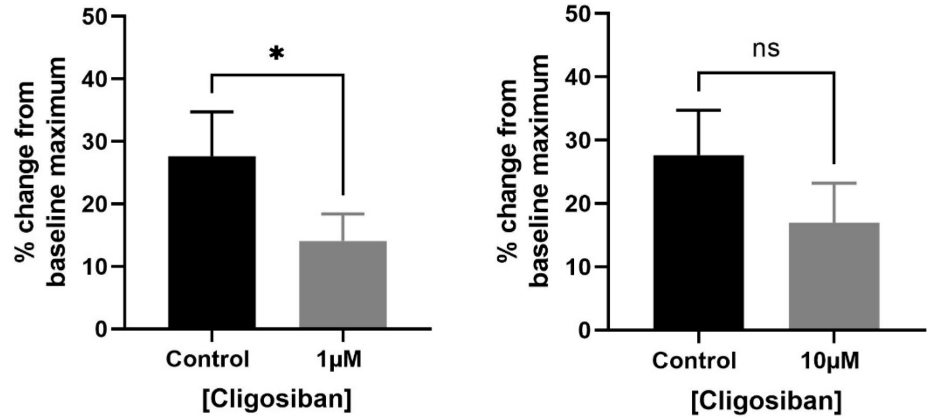

(a) (b)

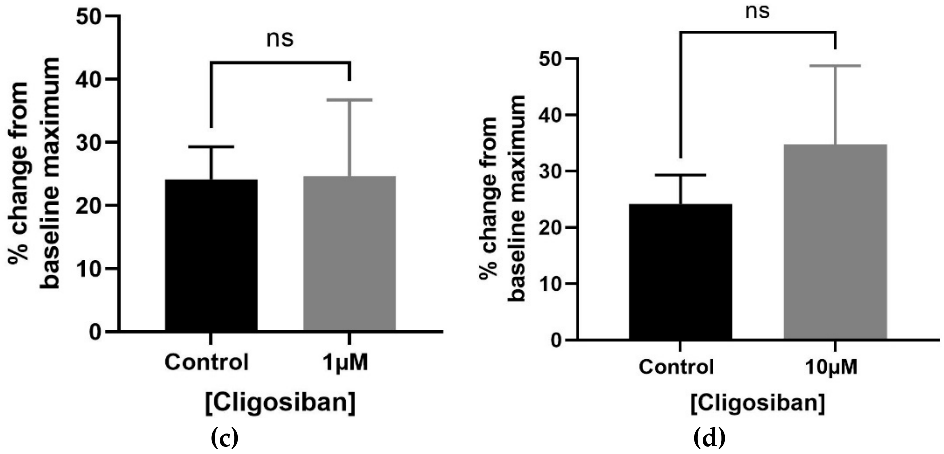

(c) (d)

Figure S3. Exogenous cligosiban (1 µM) significantly reduced the baseline maximum value, a contractile parameter of spontaneous contractions within the bladder of older (4–5 months) (a), but not the young (7–8 weeks) (c) rats, with no such effect seen at 10 µM concentration in both older (b) and young (d) rats, calculated relative to the maximum percentage of KCl (20 mM) [unpaired

t-test, n = 5, $^{*}p < 0.05$] (* denotes significant effects while ns indicates non-significant effects).

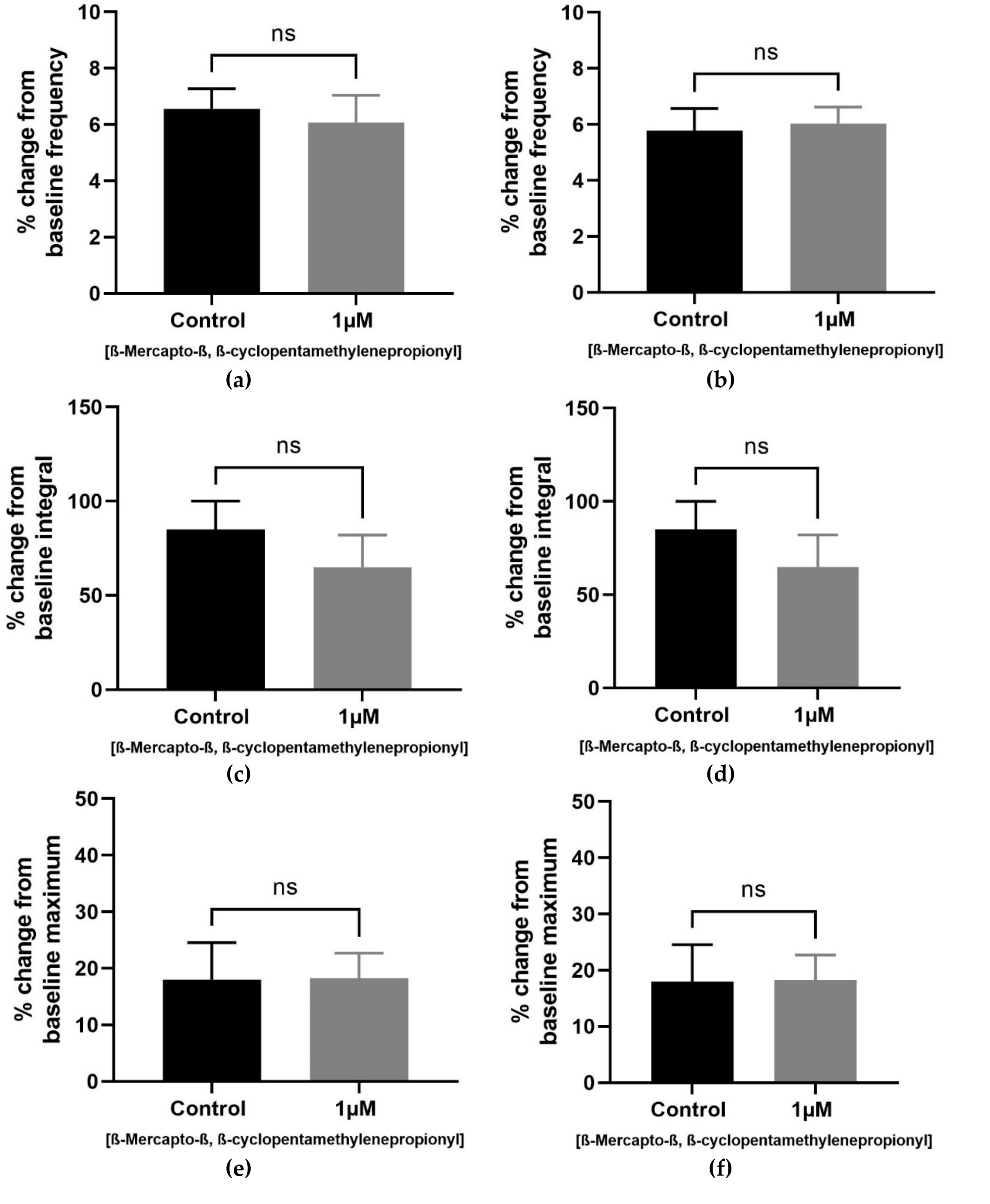

Figure S4. Effects of ßMßßC on the bladder (spontaneous contractions). Exogenous ßMßßC (1 μM) decreased the % change from (i) baseline frequency in older (a), but not young (b), (ii) baseline integral in both older (7–9 months) (c) & young (7–8 weeks) (d) rats. However, no effect was noticed on the % change from baseline maximum value in both age groups (e = old, f = young) rats [unpaired t-test, n = 5, $p \geq 0.05$] (ns indicates non-significant effect).

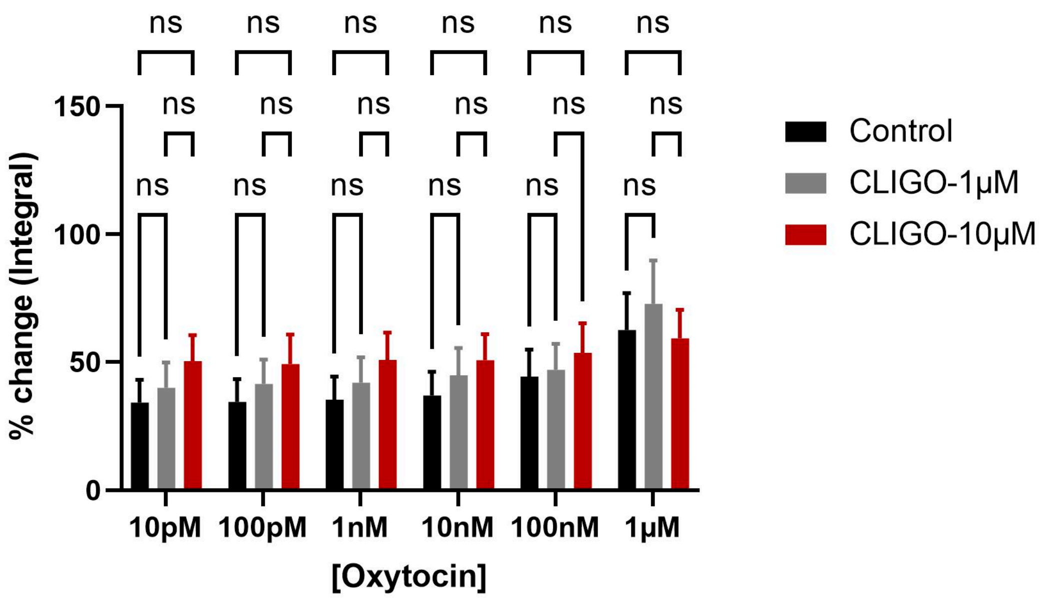

(a)

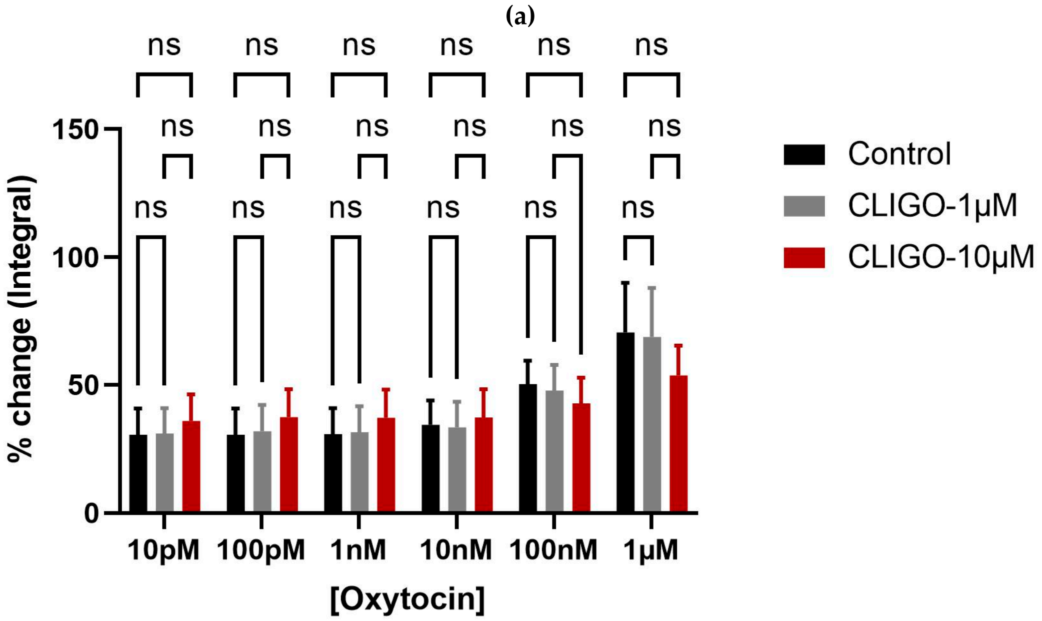

(b)

Figure S5. Application of cligosiban on bladder contractions. Cligosiban (1 µM & 10 µM) showed a non-significant effect on the oxytocin (1 µM)-induced bladder contractions within both young (7–8 weeks) (a) and older (4–5 months) (b) rats, as a percentage of change in integral (AUC) that is measured relative to the maximum percentage of KCl (20 mM) [two-way ANOVA Tukey's multiple comparison test, $p \geq 0.05$] (ns shows non-significant effect).

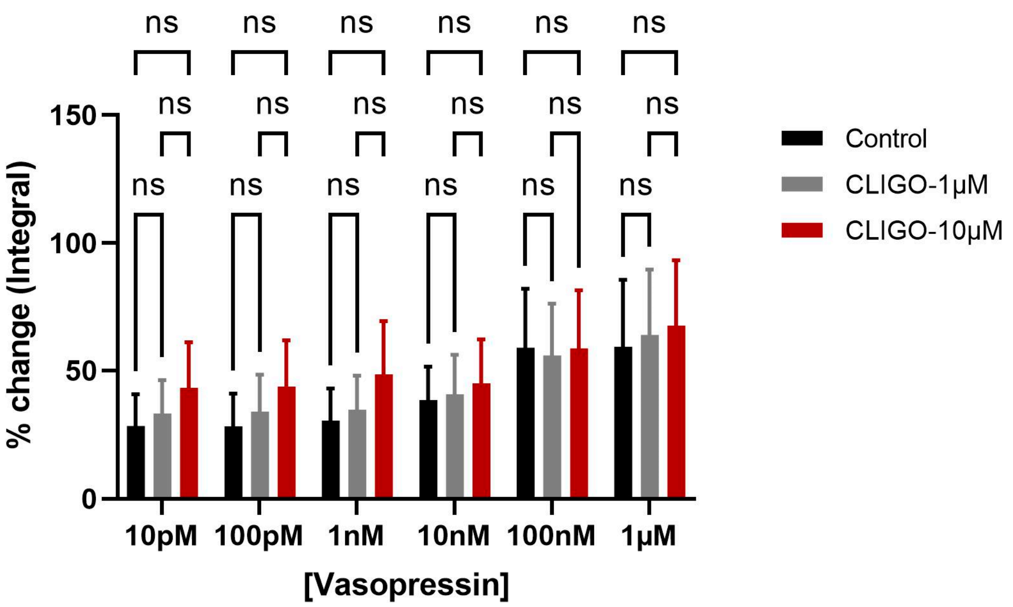

(a)

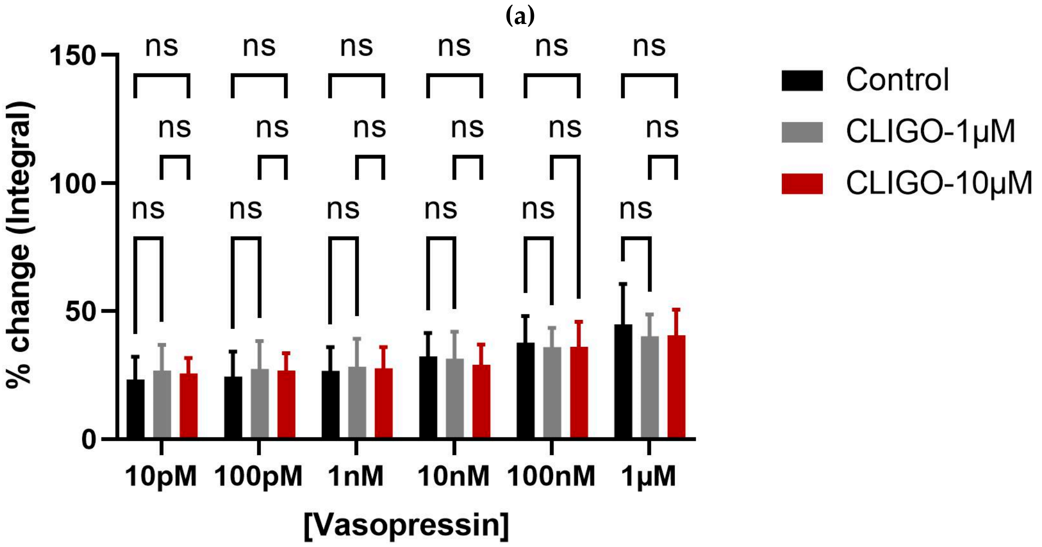

(b)

Figure S6. Effects of vasopressin on bladder contractions. Vasopressin (1 μM & 100 nM) significantly increased bladder contractions, while the cligosiban (1 μM & 10 μM) had some antagonistic effect on the % change in the integral parameter of bladder contractions and this effect was achieved at the high concentration of vasopressin (100 nM & 1 μM) in old (b), but not young (a) rats [two-way ANOVA Tukey's multiple comparison test, $p \geq 0.05$] (ns shows non-significant effect).

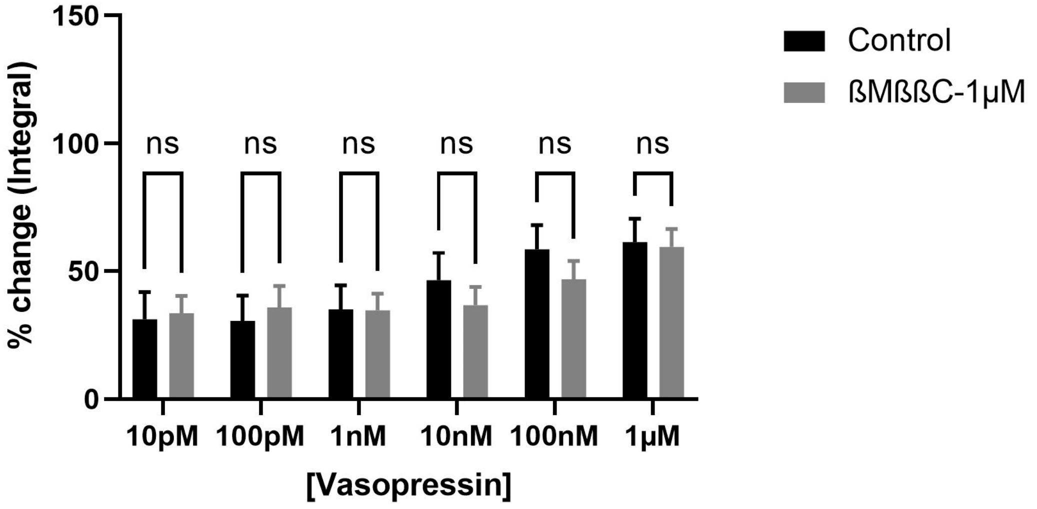

(a)

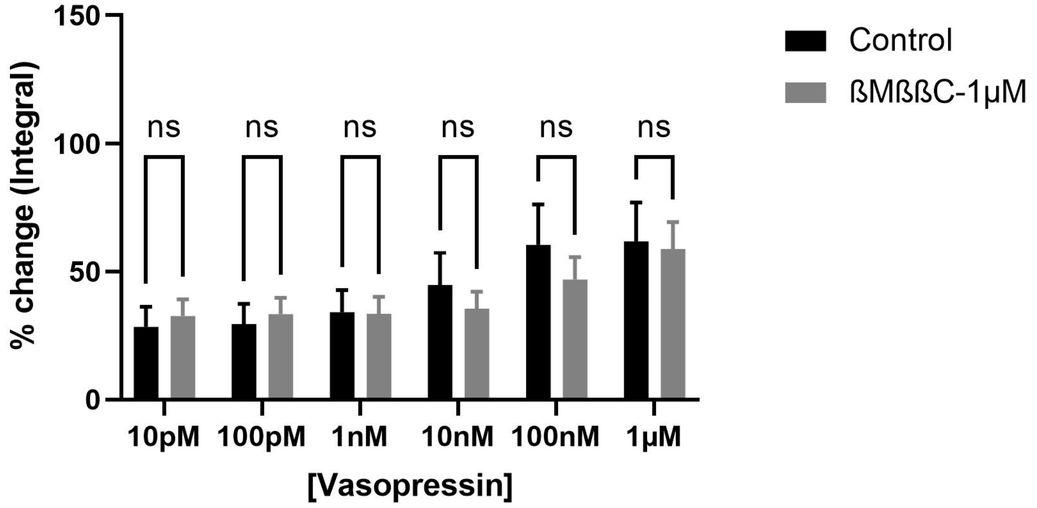

(b)

Figure S7. Vasopressin (VP) (1 µM & 100 nM) (a) showed a significant and ßMßßC (1 µM) (b) showed a trend in decreasing the % change in integral parameter of VP-induced bladder contractions in young (a) and old (b) rats, but this effect was statistically non-significant [two-way ANOVA Sidak's multiple comparison test, $p \geq 0.05$] (ns indicates non-significant effect)..
